# Using two retrotransposon-based marker systems (SRAP and REMAP) for genetic diversity analysis of Moroccan Argan tree

**DOI:** 10.22099/mbrc.2020.36390.1478

**Published:** 2020-09

**Authors:** Ouafae Pakhrou, Leila Medraoui, Bouchra Belkadi, Farid Rachidi, Hasnaa Errahmani, Mohammed Alami, Abdelkarim Filali-Maltouf

**Affiliations:** Laboratory of Microbiology and Molecular Biology, Faculty of Sciences, Mohammed V University, Rabat, Morocco

**Keywords:** Argania spinosa L., Retrotransposons, Genetic diversity, Outlier

## Abstract

The Argania is an endemic genetic resource in Morocco holding an important ecological and socio-economical benefit. However, overgrazing and overharvesting lead to a serious downturn in the number of trees. To characterize genetic diversity within and among 24 populations, represented by 240 argan trees, four combinations of SRAP primers and eight combinations of REMAP primers were used. A total of 338 REMAP and 146 SRAP markers were amplified with a polymorphism of 100%. The average polymorphism information content value was 0.20 and 0.17 for SRAP and REMAP markers, respectively. The analysis of molecular variance showed that 26% of the genetic variation was partitioned among populations. The coefficient of gene differentiation was 0.2875 and gene flow was 1.2391. The average parameter diversity was: observed number of alleles (Na)=0.729, effective number of alleles (Ne)=1.131, Shannon’s information index (I)=1.143; Nei’s gene diversity (H)=0.093 and Percentage of Polymorphic Loci=35.68. The STRUCTURE and principal coordinate analysis revealed that the *Argania spinosa* L. populations were aggregated into 2 genetic groups. To detect outlier, baysecan software was used and 21 were detected (7 under selection, 14 under balancing selection) presenting posterior probability higher than 0.79. The current results can be explored in the design of management programs and to comprehend the adaptation mechanism of Argan tree.

## INTRODUCTION

The argan tree (*Argania spinosa* L.) is the unique representative of the Sapotaceae family in Morocco, it is an endemic species of the country where it occupies the second place after the holm oak. It is widely distributed in the southwestern region of Morocco with an area of more than 900,000 ha [1]. Its main interest lies in its fruit, that gives very valuable oil for therapeutic, cosmetic and food uses [2, 3]. Also, the argan tree plays a remarkable and irreplaceable role in the ecological balance. Because of its powerful root system, it allows fighting against water and wind erosion and contributes to the maintenance of the soil. However, due to repeated droughts, excessive exploitation and insufficient natural regeneration, the argan forest has undergone alarming deterioration in less than a century. More than its half has disappeared and its average density has decreased from 100 to 30 trees/ha and even 10 trees per hectare [4]. In the face of this accelerated degradation, it is extremely important to develop adequate and effective strategies to conserve, restore and enhance this genetic resource. Therefore, the study of genetic variability proves a crucial step in understanding the state of diversity available and its structure and provides valuable information to exploit genetic diversity within an effective conservation program. 

In this investigation, we have chosen to study the genetic diversity of the natural populations of the argan tree by two molecular markers, Sequence-related amplified polymorphism (SRAP) and Retrotransposon-microsatellite amplified polymorphism (REMAP), based on the transposable elements, since they constitute a major component of plant genomes [5, 7]. SRAP is an appropriate molecular marking system for the analysis of plant genetic diversity and it has many features such as simplicity, reliability, flexibility, multiple loci detection and cost-effectiveness [8]. REMAPs exploit scattered and abundant repetitions of sequences such as retrotransposon LTRs. The association of these sequences together makes possible to amplify a series of bands (DNA fingerprints), using primers homologous to these large numbers of repeated copies. The markers generated are highly informative genetic markers [9]. These two approaches, whose effectiveness and credibility in the analysis of genetic variability have been demonstrated in many plant species, especially woody species [10, 13] will enable the accurate assessment of genetic diversity and better understand the structure of populations and contribute to the use and the effective conservation of the argan tree. In reviewing all the genetic diversity studies carried out on the argan tree using molecular markers including RFLP of chloroplast DNA [14], SSR [15], ISSR [16], AFLP [17] and IRAP [18], the present work constitutes originality. In fact, it is the first application of these two SRAP and REMAP marking strategies on the argan tree.

## MATERIALS AND METHODS

**Collection of plant materials and genomic DNA extraction: **240 genotypes were selected from 24 parts of Morocco for the REMAP and SRAP analysis (Table 1). Fresh leaves were collected from the adult tree, cleaned with moist paper towels and preserved at -80°C. Genomic DNA was extracted from 50 mg dried leaf tissue according to the ISOLATE II Plant DNA Kit (Bioline, USA). The quality and quantity of the DNA were determined by spectrophotometry using a NanoDrop 2000 (NanoDrop Technologies Inc., USA) and by visual assessment on a 1% agarose gel.

**Molecular analysis of SRAP: **The SRAP analysis was based on the protocol published by Keify and Beiki [19]. The reaction mixture contained 25 μl of 20 ng of template DNA, 1 x Taq buffer, 2.5 mM MgCl_2_, 0.2 mM dNTP, 0.25 µM primer and Taq 1 U DNA polymerase (Promega). Amplification was carried out in a Veriti 96-Well Thermal Cycler. The PCR reaction program consisted of initial denaturation at 94°C for 3 min, followed by 5 cycles of denaturation at 94°C for 1 min, annealing at 35 °C for 1 min and extension at 72°C for 1 min, followed by 35 cycles of denaturation at 94°C for 1 min, annealing at 50°C for 1 min and extension at 72°C for 1 min and a final extension at 72°C for 10 min. 

A total of 20 SRAP primer combinations were tested of which 4 primer combinations showed reproducible and distinct amplification of the loci were selected. PCR products were electrophoresed on 2% agarose gel (UltraPure-Invitrogen) for 2-3 h at 90 volts, stained with ethidium bromide and photographed under UV light using EnduroTM GDS (Labnet, USA). The SRAP images were transferred to the Gelcompar II software. Only the clear fragments in the size range of 50 bp to 2050 bp bases were marked as present. The binary data was exported and verified for further statistical analyses.

**Table 1:** Locations and sample size of the 24 populations of A. spinosa L.

**Regions**	**Bioclimate**	**Populations**	**Code**	**Code number**	**Sample size**	**Longitude**	**Latitude**	**Altitude (m)**
**Region1**	Semi-arid	**Retmana**	RT	1	10	9°19’	32°02’	72
	Semi-arid	**Ouled Lhaj**	OH	2	10	9°24’	31°56’	118
	Semi-arid	**Jbel Kourati**	JK	3	10	9°23’	31°47’	365
	Semi-arid	**Mramer**	MR	4	10	9°10’	31°38’	396
	Semi-arid	**Rbai**	RB	5	10	9°28’	31°32’	269
	Semi-arid	**Tamesrart**	TS	6	10	9°22’	31°21’	540
	Semi-arid	**Neknafa**	NK	7	10	9°33’	31°19’	242
	Semi-arid	**Ait Issi**	AI	8	10	9°22’	31°02’	980
	Semi-arid	**Tmanar**	TM	9	10	9°37’	31°00’	576
	Semi-arid	**Timzgida Oufetass**	TO	10	10	9°48’	30°00’	231
**Region2 **	Sub-humid	**Tizint'est**	TZ	11	10	8°23’	30°48’	1194
	Arid	**Imouzzer**	IZ	12	10	9°30’	30°39’	1100
	Arid	**Admine**	AD	13	10	9°21’	30°19’	83
	Arid	**Tafraout**	TA	14	10	9°03’	29°42’	900
	Arid	**Ait Baha**	AB	15	10	9°13’	30°06’	490
	Arid	**Mnizla**	MN	16	10	9°05’	30°33’	256
	Arid	**Oulcadi**	OL	17	10	8°28’	30°17’	1235
	Arid	**Aoulouz**	AO	18	10	8°06’	30°37’	788
	Arid	**Doutana**	DT	19	10	9°13’	30°43’	1194
**Region3**	Arid	**Lakhssas**	LA	20	10	9°43’	29°24’	954
	Saharan	**Guelmim**	GU	21	10	10°06’	29°06’	351
	Saharan	**Assa Zag**	AZ	22	10	9°25	28°25’	328
**Region4**	Sub-humid	**Oued Grou**	OG	23	10	6°22’	33°27’	412
	Sub-arid	**Benié Snassen**	BS	24	10	2°34’	34°35’	246

**Molecular analysis of REMAP: **Eight REMAP primer combinations were selected for further analysis. The REMAP-PCR was performed in 25 µl reaction mixtures containing: 8ng/µl of DNA, 2 µM of each primer, and 13 µl MyTaq HS Mix (BIOLINE). PCR cycles started with an initial denaturation step at 94 °C for 1 min followed by 35 cycles of denaturation at 95 °C for 15s, annealing at the specific annealing temperature for 15 s and extension at 72 °C for 10 min. Gel electrophoresis and notation bands were performed as described for the SRAP. The fragments in the size range of 50 bp to 1500 bp bases were marked as present.

**Data analysis: **Genotypic data was used to evaluate the efficacy of each primer used in each marker system. The polymorphic information content (PIC) was determined according to Roldán-Ruiz et al., [20], the marker index (MI) was calculated using the formula given by Powell et al., [21], while the resolving power (Rp) was calculated according to Prevost and Wilkinson [22] based on the distribution of alleles of all 240 genotypes of *A. spinosa* L. 

**Genetic diversity and population structure analyses: **The combined binary data matrix of markers SRAP and REMAP was used to estimate genetic differentiation (Gst) and gene flow (Nm) using POPGENE v.1.32 [23]. The genetic diversity parameters such as the percentage of polymorphic loci (%P), the Shannon information index (I), the genetic diversity of Nei (H), the effective number of alleles (Ne) and the number of alleles observed (Na), were estimated using GenAlEx version 6.5 [24]. The same software was also used to estimate the partition of genetic variance among and within populations by the analysis of molecular variance (AMOVA) and to ordinate relationships among populations based on genetic distance matrix by Principal Coordinates Analysis (PcoA).

The population structure was carried out based on the REMAP and SRAP genotypes using the Bayesian clustering model implanted in STRUCTURE software version 2.3 [25]. The number of population K was tested from 1 to 15 with a burn-in time period of 10000 followed by 100000 Markov Chain Monte Carlo (MCMC) iterations. The program was run 20 times for each K number based on admixture model and on correlated allele frequencies. The highest value of ΔK, representing the most likely number of clusters [26], was determined using Structure Harvester [27].

The candidate loci in the selection was identified using a Bayesian approach based on Fst described by Beaumont and Balding [28] implemented in BayeScan software version 2.1 [29]. On the basis of dominant marker data, the analysis directly estimates the probability that a locus is under selection by calculating a ratio of the posterior probabilities of under selection models or neutral models [30]. The default parameters were used: 20 pilot runs, a burn-in period of 50,000 iterations and 5,000 output iterations with a thinning interval of 10. The outlier markers were selected starting from 0.79 posterior probabilities. To eliminate the false positives among the outliers found, the rate of false discovery (FDR) was calculated. Thus, BayeScan program determines a q-value, which is the FDR analogue of the p-value. The loci with a q-value over 1% were retained as outlier.

## RESULTS

To reveal the genetic diversity and outlier markers for 240 individuals of argan tree two markers (SRAP and REMAP) were used in this study. Four SRAP out of 20 primers and 8 REMAP gave polymorphism and were selected for data analysis. Four selected SRAP markers amplified a total of 147 amplicons ranged from maximum 45 (ME-1/EM-4) to minimum 32 (ME-2/EM-4) with an average of 36.75 bands displaying an overall polymorphism of a 100%. The polymorphism information content (PIC) showed an average of 0.20, the highest PIC was 0.21 showed by ME-2/EM-4 and ME-4/EM-4. The mean of resolving power (Rp) and markers index (MI) was 10.74 and 7.33 respectively while the highest value Rp (12.51) was generated by ME-2/EM-6 and by ME-1/EM-4 for MI (11.53). The eight REMAP primer combinations also showed a high polymorphism (100%) amplifying 338 polymorphic bands with an average of 42.25. The REMAP primer combinations IRAP3/ISSR2 and IRAP1/ISSR2 represent the lowest (34) and the highest (52) values, respectively, of the labeled alleles. The PIC of the REMAP markers varied from 0.20 for the combination of primers IRAP3/ISSR2 and IRAP2/ISSR2 to 0.14 for the primer combination IRAP4/ISSR1. The highest value of the resolving power (10.20) and the marker index (8.30) were obtained with primer combination IRAP1/ISSR2. A summary of the number of bands, %P, PIC, Rp and MI reproduced by the 4 SRAP and the 8 REMAP is shown in Table 2.

**Table 2:** Degree of polymorphism and polymorphic information content, for REMAP and SRAP primers in 240 individuals of Argan tree

**Primers**	**Sequence (5’ – 3’)**	**Tm** ^a^	**TF** ^b^	**%P** ^c^	**PIC** ^d^	**MI** ^e^	**Rp** ^f^
**SRAP**							
ME-1/EM-4	TGAGTCCAAACCGGATA GACTGCGTACGAATTTGA		45	100	0.20	8.96	11.53
ME-2/EM-4	TGAGTCCAAACCGGAGC GACTGCGTACGAATTTGA		32	100	0.21	6.68	9.43
ME-2/EM-6	TGAGTCCAAACCGGAGC GACTGCGTACGAATTGCA		37	100	0.18	6.68	12.51
ME-4/EM-4	TGAGTCCAAACCGGACC GACTGCGTACGAATTTGA		32	100	0.21	7.01	9.50
Total			146		0.80	29.33	42.98
Mean			36.75		0.20	7.33	10.74
**REMAP**							
IRAP1/ISSR1	GATAGGGTCGCATCTTGGGCGTGAC(AG)_8_CC	53	47	100	0.17	7.87	9.45
IRAP1/ISSR2	CTCGCTCGCCCACTACATCAACCGCGTTTATT(GA)_8_CT	50	52	100	0.16	8.30	10.20
IRAP2/ISSR1	CTCGCTCGCCCACTACATCAACCGCGTTTATT(AG)_8_CC	53	44	100	0.16	7.03	8.55
IRAP2/ISSR2	CTCGCTCGCCCACTACATCAACCGCGTTTATT(GA)_8_CT	50	40	100	0.20	7.84	10.21
IRAP3/ISSR1	TGTTTCCCATGCGACGTTCCCCAACA(AG)_8_CC	53	43	100	0.15	6.29	7.62
IRAP3/ISSR2	TGTTTCCCATGCGACGTTCCCCAACA(GA)_8_CT	50	34	100	0.20	6.73	8.81
IRAP4/ISSR1	TTGCCTCTAGGGCATATTTCCAACA(AG)_8_CC	53	43	100	0.14	5.89	6.82
IRAP4/ISSR2	TTGCCTCTAGGGCATATTTCCAACA(GA)_8_CT	50	35	100	0.18	6.34	7.94
Total			338		1.35	56.37	69.59
Mean			42.25		0.17	7.05	8.70

^a^Annealing temperature; ^b^Total number of fragments; ^c^Percent polymorphism; ^d^Polymorphic information content; ^e^Marker Index; ^f^Resolving power.

The average number of alleles observed (Na), effective number of alleles (Ne), Shannon's information index (I) and Nei's genetic diversity (H) in the argan tree populations were 0.729, 1.131, 0.143 and 0.088, respectively. The highest values of diversity parameters were observed in Mramer population (Na= 0.864, Ne=1.170, I=0.178, H= 0.117, P= 42.15%) (Table 3). The results of AMOVA analysis revealed that variation within populations (74%) was significantly higher than that among populations (26%). Similarly, The Gst value was 0.2875 and the gene flow was 1.2391 (Table 4).

**Table 3:** Genetic diversity statistics in 24 populations of A. spinosa L. Ten individuals were sampled from each population

**Populations**	**Na ** ^a^	**Ne ** ^b^	**I ** ^c^	**H ** ^d^	**P ** ^e^
**Retmana**	0.653	1.114	0.127	0.082	31.82%
**Ouled Lhaj**	0.829	1.154	0.164	0.107	40.08%
**Jbel Kourat**	0.684	1.139	0.145	0.096	33.47%
**Mramer**	0.864	1.170	0.178	0.117	42.15%
**Rbai**	0.709	1.137	0.145	0.095	34.50%
**Tamesrart**	0.733	1.159	0.162	0.109	35.74%
**Neknafa**	0.878	1.159	0.175	0.114	42.77%
**Ait Issi**	0.837	1.140	0.156	0.100	41.12%
**Tmanar**	0.568	1.093	0.107	0.068	28.10%
**Timzgida Oufetass**	0.729	1.118	0.134	0.085	36.16%
**Imouzzer**	0.659	1.126	0.134	0.088	32.64%
**Admine**	0.746	1.125	0.142	0.091	36.78%
**Tafraout**	0.671	1.105	0.121	0.077	32.85%
**Ait Baha**	0.824	1.149	0.165	0.107	40.91%
**Mnizla**	0.777	1.154	0.161	0.106	38.22%
**Tizint** **'** **est**	0.820	1.134	0.152	0.096	40.50%
**Aoulouz**	0.816	1.145	0.156	0.101	39.88%
**Doutana**	0.841	1.160	0.172	0.112	41.53%
**Lakhssas**	0.795	1.130	0.149	0.095	39.26%
**Oulcadi**	0.680	1.108	0.124	0.079	32.85%
**Guelmim**	0.568	1.098	0.111	0.071	27.69%
**Assa Zag**	0.446	1.074	0.082	0.053	21.49%
**Oued Grou**	0.775	1.141	0.150	0.098	37.40%
**Benié Snassen**	0.595	1.113	0.119	0.078	28.51%
**Mean**	0.729	1.131	0.143	0.093	35.68%

^a^Observed number of alleles; ^b^Effective number of alleles; ^c^Shannon’s information index; ^d^Nei’s (1973) gene diversity; ^e^Percentage of Polymorphic Loci.

**Table 4:** Analysis of molecular variance (AMOVA) using REMAP and SRAP markers for 24 populations of A. spinosa L

**Source**	**Df** ^a^	**SS** ^b^	**MS** ^c^	**VC** ^d^	**% VC** ^e^	**Gst** ^f^	**Nm** ^g^
**Among populations**	23	3415.217	144.488	11.529	26%	0.2875	1.2391
**Within populations**	216	7171.400	33.201	33.201	74%		
**Total**	239	10586.617		44.730	100%		

^a^Degrees of freedom; ^b^Sum of squares; ^c^Mean of squares; ^d^Variance component; ^e^%Variance component; ^f^Coefficient of genetic differentiation; ^g^Estimate of gene flow.

**Figure 1:**
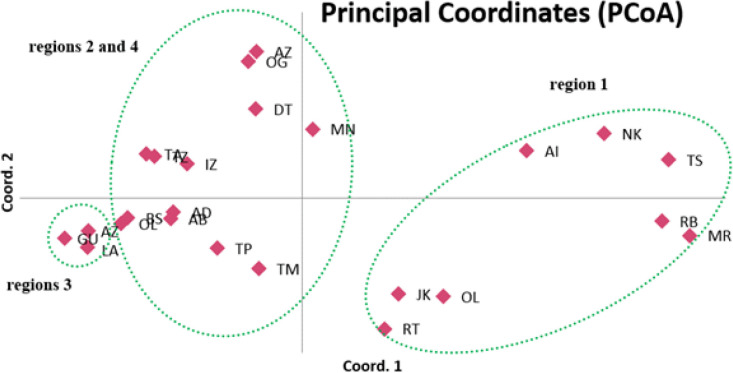
Principal coordinate analysis (PCoA) among 240 individuals of A. spinosa L. Populations codes see Table 1

To visualize the relationships inter populations and elucidate the genetic structure of 24 populations of argan tree analysis PCOA and STRUCTURE were used. The three coordinates of PCoA analysis displayed 51.49 % of total variation. The first axis explained 10.95 % of the variance, while the second and the third ones displayed 18.48% and 22.51% of the variance respectively (Fig. 1).

**Figure 2:**
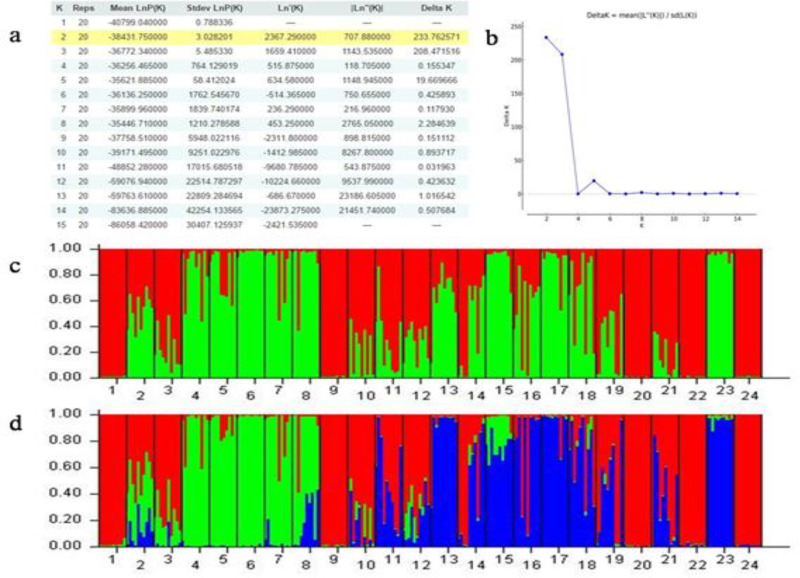
The results of genetic structure analyses by STRUCTURE software. a) Table output of the Evanno method results implemented in STRUCTURE HARVESTER. Yellow highlight shows the largest value in the Delta K column suggesting that the most likely number of *A. spinosa* L. genetic groups is k=2 based on 24 populations representing the global natural distribution of argan tree in Morocco. b) K as per Evanno et al., [26] plotted against the number of genotype clusters (K), where K = 2 is the best fit of the populations followed by k=3. c) STRUCTURE output for K= 2 and d) K = 3. For populations numbers see Table 1

A bayesian analysis was performed with K value (possible cluster number) ranging from 1 to 15. The second order statistics (ΔK) developed by Evanno et al., [26] showed a clear maximum for K = 2 (ΔK = 233.762571) indicating that the 24 populations could be grouped into two genetic clusters (Fig. 2). The first cluster mainly consisted of 14 populations (2, 3, 4, 5, 6, 7, 8, 13, 14, 15, 16, 17, 18, 19 and 23) and the second cluster mainly consisted of 10 populations (1, 9, 10, 11, 12, 20, 21, 22 and 24). At the same time, the second largest ΔK at K = 3 was much larger than the remaining values. On the basis of K=3, the first cluster was separated into two new genetic clusters. Some populations formed a clear separation indicating high differentiation.

## DISCUSSION

The efficiency of genetic diversity analysis depends largely on the polymorphism detected by molecular markers. In this study, SRAP and REMAP markers were used to assess genetic diversity among 240 argan genotypes representing 24 natural ecosystems. These markers are highly polymorphic due to their abundance in plants genomes and their dynamic nature [5, 7]. Their efficiency to assess genetic diversity has been proven in different studies such as [10, 11, 13, 31, 34]. Also, many other studies suggest that retrotransposon-based molecular markers are more useful and more informative than other markers such as RAPD, AFLP or SSR [35, 37]. Out of all primer combinations tested, 4 SRAP and 8 REMAP amplified good polymorphism and scorable banding patterns. These primers were selected to study genetic diversity of argan tree. 

Despite the fact that the mean number of polymorphic bands amplified by REMAP was higher to that observed using the SRAP, the two markers have revealed a high level of polymorphism (100%). In addition, evaluation of informativeness and discriminatory power of the two marker systems by calculating the PIC, Rp and MI has shown higher mean values for SRAP compared to REMAP. The result found in this study is comparable to other studies suggesting that SRAP is the most efficient technique to reveal informative bands [38, 39]. 

However, the combined use of different approaches could give more reliable information about genetic diversity [40, 41]. In fact, the two markers used in this study differ by their target genome region. The REMAP technique relies on the amplification of sequence between a microsatellite and retrotransposon [42] while the SRAP technique detects polymorphisms by amplifying the open reading frames (ORFs) [43]. 

Consequently, the combined data will enable good genome coverage. Thus, the results of the two molecular techniques were combined and the data of 484 loci was obtained. Based on these data, the analyses of the molecular variance (AMOVA) was used to evaluate the genetic variability in different populations and revealed that most of the variance was attributed to the divergence within populations. This has been confirmed by the coefficient of genetic differentiation (Gst = 0.2875). These results are consistent with those found in other research studies focusing on genetic diversity of argan tree using IRAP-ISSR and AFLP markers [17, 18] and can be explained firstly by the habitat of argan trees naturally fragmented. This distribution leads to a high level of genetic differentiation among populations of argan tree in comparison with other forest trees [44]. Secondly, the argan tree can reproduce with double pollination system by anemophily or entomophily [44]. Thus, the individual exchange genes with its closest creating a high gene flow between populations geographically close. This result was confirmed by PCoA and revealed that the majorities of the populations belonging to the same region have been regrouped except the populations of region 4 (Beniznsaen and Oued Grou) which can be a result of recent dispersion probably caused by human activities. The bayesian analysis divided all of the 24 populations into two genetic pools (K=2). This structuration can result from different factors, either historic, geographic or genetic [47]. Moreover, the populations of argan tree exist in Morocco under different climates (Sub-humid, arid, semi-arid and Saharan) which can cause the variations of flowering period and as a consequence preventing gene flow between different populations.

The parameters of genetic diversity were also calculated from the combined data of the two markers and the highest values were found by Mramer population. This population should have priority for conservation. In general, the revealed genetic diversity was higher than the values found in endangered or endemic forest species. 

However, the values found in this study were higher than those found by Pakhrou et al., [18] using combined data of IRAP and ISSR markers for assessing the genetic diversity of the same populations. This comparison reveals that the indices of genetic diversity can vary according to the techniques used and confirm that retrotransposon-based molecular markers were more useful to detect genetic variations. Hence, the comparison of the ability of each marker to assess genetic diversity of argan tree seems to be a good perspective and will enable a better understanding of their efficiency and utility and could also provide valuable information.

The combined data has been also the subject to detect outlier. Although retrotransposon-based molecular markers are assumed to be neutral markers. 

These markers generate a good number of polymorphic loci, which also can match gene encoding specific proteins. Recently, many dominants markers have been used to detect outlier such as SRAP and ISSR [48-50]. In the present investigation, the software BayeScan was selected for its performance to detect outlier and to reduce the number of false positive [51, 52] and twenty-one outliers were detected (7 under selection and 14 under balancing selection). Our results are in line with the result of other studies using dominant markers to detect outlier and reject the null hypothesis based on neutral allele model [45]. The basis genetic found in the present study can be used to investigate the adaptability of argan tree to the variations of the climate, especially by analyzing the correlation between climatic variable (temperature and precipitation) and adaptive loci, and to predict the response of argan tree to future climate changes. 

The study of genetic diversity is one of the crucial and essential steps for effective conservation and preservation of argan forest. This is the first report of genetic diversity study of *Argania spinosa* L. using REMAP and SRAP markers. A total of 240 tree of argan trees were collected from 24 regions representing its natural distribution. The results found in the present study indicate that high genetic differentiation was found mainly within population and showed a clear genetic structure which grouped the 24 populations into two genetic groups. this study demonstrated that SRAP and REMAP markers provide appropriate information for genetic structure of argan tree and could be used as an efficient tool for the detection the molecular relationships of trees. The data can be explored to study adaptation mechanisms of argan tree.
